# Cardioprotective Effect of Nec-1 in Rats Subjected to MI/R: Downregulation of Autophagy-Like Cell Death

**DOI:** 10.1155/2021/9956814

**Published:** 2021-07-12

**Authors:** Liang Wang, Xuebai Lv, Jue Tian, Xiaoliang Wang, Ye Wu, Hui Rong Liu

**Affiliations:** ^1^Department of Cardiology, Peking University International Hospital, Beijing 030001, China; ^2^Third Medical Center, The General Hospital of the People's Liberation Army, Beijing 102206, China; ^3^Department of Physiology, Shanxi Medical University, Taiyuan 030001, China; ^4^Department of Pathophysiology, Ningxia Medical University, Yinchuan, Ningxia 750004, China; ^5^Department of Pathophysiology, School of Basic Medical Sciences, Capital Medical University, Beijing 100029, China; ^6^Beijing Key Laboratory of Metabolic Disorders Related Cardiovascular Disease, Capital Medical University, Beijing 100069, China

## Abstract

**Objective:**

Necrostatin-1 (Nec-1), an inhibitor of necroptosis, has been reported to protect against myocardial ischemia-reperfusion (MI/R) injury. However, the contribution of the potential antinecroptotic effect of Nec-1 on its infarct limitation and cardiac function improvement effects after MI/R has not been investigated.

**Methods:**

The present study investigated the effect of Nec-1 on myocardial infarct size, necroptosis, and cardiac functional recovery in rats subjected to myocardial ischemia-reperfusion (MI/R 30 min/12, 24, 48, and 72 h).

**Results:**

The study showed that Nec-1 might reduce myocardial cell death and maintain myoarchitectonic integrity, consequently inhibiting the reactive fibrosis process in rats in myocardial ischemia/late reperfusion. Moreover, the administration of Nec-1 (0.6 mg/kg) at the onset of reperfusion significantly reduced the release of creatine kinase and downregulation of autophagy within 24 h after reperfusion, and there was a significantly positive correlation between them.

**Conclusion:**

These results suggest that antinecroptosis treatment may improve the clinical outcomes of patients with ischemic heart disease.

## 1. Introduction

Ischemic heart disease is the most severe cause of death in developed countries [1]. Early reperfusion after coronary obstruction is the most effective means of limiting ischemic myocardial injury. However, abundant evidence suggests reperfusion may cause additional cell death called reperfusion injury [2, 3]. It has long been recognized that the loss of cardiomyocytes, an increase in replacement, and reactive fibrosis resulting in scar formation lead to cardiac dysfunction after myocardial ischemia-reperfusion (MI/R) [4, 5]. Blocking the signal transduction leading to cell death significantly reduces myocardial infarct size and improves myocardial functional recovery after reperfusion [4, 6, 7].

Growing evidence from animal experiments and clinical observations indicates that myocardial infarction after ischemia and reperfusion is caused by necrosis, a traditional cell death pathway, and apoptosis, a gene-controlled programmed cell death. Although the contribution of necrosis to infarct size is considerable [8, 9], apoptosis as programmed cell death is generally considered to be more controllable than necrosis. Recently, numerous studies have substantiated that apoptosis is not an exclusively programmed cell death [10–13]. For example, autophagic cell death as a necrosis-like cell death has been confirmed as a novel programmed cell death (termed type II programmed cell death) [11, 12, 14].

Type II programmed cell death is characterized by a massive cytosolic autophagosome, which can be suppressed by downregulating the formation of autophagosomes [15]. Autophagy was considered a self-salvaged cell mechanism in response to the serious condition, which has a protective role within a certain threshold. Exceeding the threshold will result in cell death [16, 17]. However, recent studies have found that autophagic cell death induced by MI/R could not be inhibited by blocking the autophagy pathway [18–20]. Therefore, this research considered that an upstream mechanism of autophagy might be a critical event in mediating cell death during MI/R, but a lack of suitable tools limited further investigation.

Degterev et al. discovered a small tryptophan-based molecule called Necrostatin-1 (Nec-1), which can specifically inhibit a nonapoptosis pathway from necrosis-termed necroptosis and protect the cerebral cortex against ischemia/reperfusion (I/R) injury. Furthermore, they showed that necroptosis was a delayed process, and autophagy could be induced by necroptosis in cerebral I/R injury [13]. Some studies have discovered that neurons and cardiomyocytes, as terminally differentiated cells, are more sensitive to autophagy than other cell types [21, 22]. Therefore, the effects of Nec-1 on the heart and brain may be similar and could potentially have a significant therapeutic value to MI/R injury. It has recently been demonstrated that Nec-1 had cardioprotective effects in 2 and 4 h reperfusions after 30 min of myocardial ischemia [23, 24]. These results strongly suggest that necroptosis occurring in ischemic/reperfused cardiomyocytes may cause cardiac cell death and that Nec-1 may exert its infarct reduction effect partially through an antiautophagic effect. However, to date, the contribution of the potential antinecroptotic effect of Nec-1 on its infarct limitation and cardiac function improvement effects after myocardial I/R has not been directly investigated.

Accordingly, the aims of the present experiment were (1) to investigate whether administration of Nec-1, a specific inhibitor of necroptosis, may reduce autophagy-like cell death induced by MI/R and thus contribute to its infarct reduction and myocardial functional recovery after reperfusion *in vivo*, and, if so, (2) to determine the influence of myoarchitectonic necessary changes on antinecroptosis effects associated with the administration of Nec-1 in the setting of myocardial ischemia and reperfusion.

## 2. Materials and Methods

### 2.1. Animal Experiment Protocol

The experiments were performed in adherence to the Guide for the Care and Use of Laboratory Animals protocol, published by the Ministry of the People's Republic of China (issued June 3, 2004) and approved by the Institutional Committee on Animal Care (Ethical Number: 2017-025 (BMR)). The Sprague-Dawley rats used in the present study were obtained from the Bill Animal Farm (Mianyang, Sichuan province, China). The rats were housed in pathogen-free conditions at approximately 20°C and were exposed to a light cycle of 12 h light and 12 h dark. They were fed rat chow and water *ad libitum* throughout the study period. An acclimation period of at least one week was provided before initiating the experimental protocol.

### 2.2. Myocardial I/R Protocol

Male Sprague-Dawley rats (200–285 g) were anesthetized with 0.3 g/kg i.p. of 10% chloraldurate and ventilated with a small animal respirator. A limb Lead II electrocardiogram (ECG) was recorded. Myocardial ischemia (MI) was produced by temporarily exteriorizing the heart through a left thoracic incision and placing a 6-0 silk suture slipknot around the left anterior descending coronary artery (LAD). After 30 min of MI, the slipknot was released, and the myocardium was reperfused (R) for 12, 24, 48, and 72 h. Rats were randomized to receive either the vehicle (0.05% DMSO) or Necrostatin-1 (0.6 mg/kg, Sigma-Aldrich, Inc., USA) via the caudal vein at the onset of reperfusion. Sham-operated control rats (sham MI/R) underwent the same surgical procedures except that the suture placed under the left coronary artery was not tied. At the end of the R period, the heart was quickly excised, and the I/R cardiac tissue was isolated and processed according to the procedures described below.

### 2.3. Determination of Cardiac Histology

At 72 h after reperfusion, myocardial samples were fixed in 10% formalin and embedded in paraffin and transversely sectioned off (5 *μ*m). Masson's trichrome stain (Sigma-Aldrich, Inc., USA) was used to trace the areas of the myocardial infarct zone and the extent of collagen deposition and organization of collagen fibrils at 72 h after reperfusion.

After reperfusion, samples at 48 h were fixed in 2.5% glutaraldehyde in 0.1 M phosphate buffer (pH 7.4) for 2 h at 4°C. The specimens were then rinsed in buffer and postfixed with 1% osmium tetroxide in the phosphate buffer for 2 h at 4°C. After being dehydrated with a graded series of dehydrating acetone, the specimens were infiltrated with Epoxy resin 618. Ultrathin sections (50 *μ*m thickness) were cut using the LKB Ultramicrotome IV after polymerization at 60°C, stained with uranyl acetate and lead citrate solution, and observed using a 100-CX transmission electron microscope. The relevant calculation formula was myocardial infarction = LV infarct zone/LV area × 100%.

### 2.4. Determination of Cardiac Function Injury

MI/R-induced cardiac dysfunction was monitored 10 min before ischemia and 0 min, 12, 24, 48, and 72 h after MI/R. The left ventricular pressure (LVD), including left ventricular systolic, diastolic, and end-diastolic pressures (LVSP, LVDP, and LVEDP, respectively), was digitally processed via a hemodynamic analyzing system (PowerLab Hardware, ADInstruments). Heart rates (HR) and maximal positive and negative values of the instantaneous first derivative of LVP (+dp/dtmax and −dp/dtmax, respectively) were derived from computer algorithms. The postischemic recovery of cardiac function was expressed as a percentage of the preischemic value.

### 2.5. Serum Creatine Kinase Level Assay

Blood samples were collected from the abdominal aorta at different time points (12, 24, 48, and 72 h) after reperfusion from rats treated with the vehicle and Nec-1 and centrifuged at 1,000 × g for 10 min. The serum was stored at −70°C until used. According to the supplier's instructions, serum concentrations of creatine kinase (CK) were determined using a diagnostic kit (Jiancheng, Nanjing, China).

### 2.6. Western Blotting Assay

The expression of MAP-LC3*β* (microtubule-associated protein light chain 3*β*, LC3*β*) was examined by western blot. Proteins from a tissue homogenate were separated by SDS-PAGE and electroblotted onto nitrocellulose. The blots were blocked overnight in TBS with 3% BSA (bovine serum albumin) and then probed with rabbit anti-LC3*β* antiserum (1 : 3,000, Santa Cruz sc-28266) at 4°C overnight while agitated. The blots were washed once in TBST (TBS with 0.05% Tween 20) and twice for 5 min in TBS followed by incubation for 1 h at room temperature with HRP-conjugated Goat Anti-Rabbit IgG Antibody (1 : 3,000, Zhongshan Jinqiao, China). The blots were washed three times for 5 min in TBST and developed with a chemiluminescence detection kit (Pierce). The immunoblotting was scanned with an HP scanner, and the blot densities were analyzed with Image-Pro Plus 5.0 software. The relevant calculation formula was myocardial infarction = LV infarct zone/LV area × 100%.

### 2.7. Statistics

Results are expressed as a mean ± standard deviation. The statistics were calculated using a two-group *t*-test (unpaired, one- or two-sided). The correlation between the two variables was analyzed by single linear regression analysis with SPSS 15.0 statistics software. Values of *P* < 0.05 were considered to be statistically significant.

## 3. Results

### 3.1. Effect of Nec-1 in Late Reperfusion after Myocardial Ischemia

**Figure 1:**
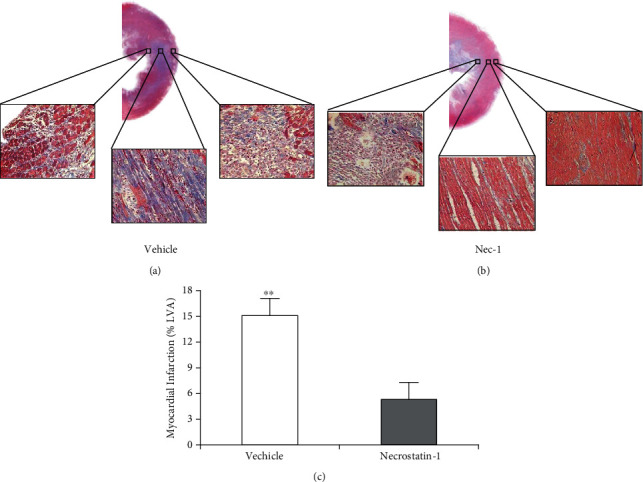
Nec-1 protects adult rats from myocardial ischemia/reperfusion injury. (a, b) Representative Masson's trichrome staining in adult rat hearts subjected to ischemia for 30 min and reperfusion for 72 h, respectively, treated with DMSO or Nec-1 at the onset of reperfusion. Reactive fibrosis for myocardial injury is stained in blue and expands from the endocardium to the epicardium. In contrast with the control, the administration of Necrostatin-1 can obviously reduce the extent of myocardial injury from the endocardium to the epicardium. (c) Graph shows myocardial infarct size measured by Masson's trichrome staining at 72 h of reperfusion. All data were expressed as mean ± SD; *n* = 10 for the vehicle group and *n* = 9 for the Nec-1 group. Scale bar, 1 mm and 40 *μ*m. ^∗∗^*P* < 0.01 Nec-1 group vs. vehicle group. LVA: left ventricular area.

The histomorphological changes and myocardial infarct size at 72 h after reperfusion were examined to determine the effects of Nec-1 in rats subjected to myocardial ischemia and late reperfusion. In anesthetized S-D rats subjected to I/R protocol, the Nec-1 (0.6 mg/kg) that was administered at the onset of reperfusion significantly reduced reactive myocardial fibrosis from the endocardium to the epicardium and the infarct size from 15.1 ± 1.7% (vehicle group) to 5.3 ± 2% (*P* < 0.01; Figure 1) by Masson's trichrome stains [5].

**Figure 2:**
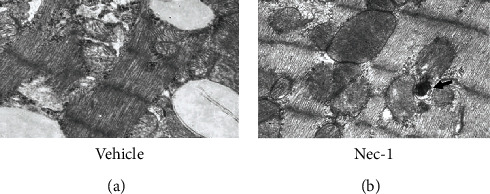
Ultrastructure of the myocardial infarct area under a transmission electron microscope at 48 h reperfusion after ischemia (x8000). (a) Electron micrographs of hearts subjected to I 30 min/R 48 h and treated with DMSO show massive vacuoles in myocardial tissue. (b) Electron micrographs of hearts subjected to I 30 min/R 48 h and treated with Nec-1+DMSO show a few autophagosomes, but the myocardial ultrastructure has more integrity than the vehicle group. Arrows indicate autophagosomes. *n* = 10 for the vehicle group and *n* = 9 for the Nec-1 group.

Through observing LV tissue at 48 h after reperfusion with a transmission electron microscope, it was further found that myocardial ultrastructure in the vehicle group had been severely damaged; cardiomyocytes were disintegrated and had formed massive vacuoles but almost no autophagosomes. Inversely, treatment with Nec-1 can maintain myocardial ultrastructural integrity and markedly reduce the loss of cardiomyocytes. However, there were a few autophagosomes with diffuse distribution in some cardiomyocytes (Figure 2). Thus, Nec-1 can not only salvage left ventricular (LV) tissue from ultimate death but also inhibit LV remodeling processes by reducing myocardial cell death.

### 3.2. Cardiac Functional Assessment at Different Time Points after I/R *In Vivo*

**Figure 3:**
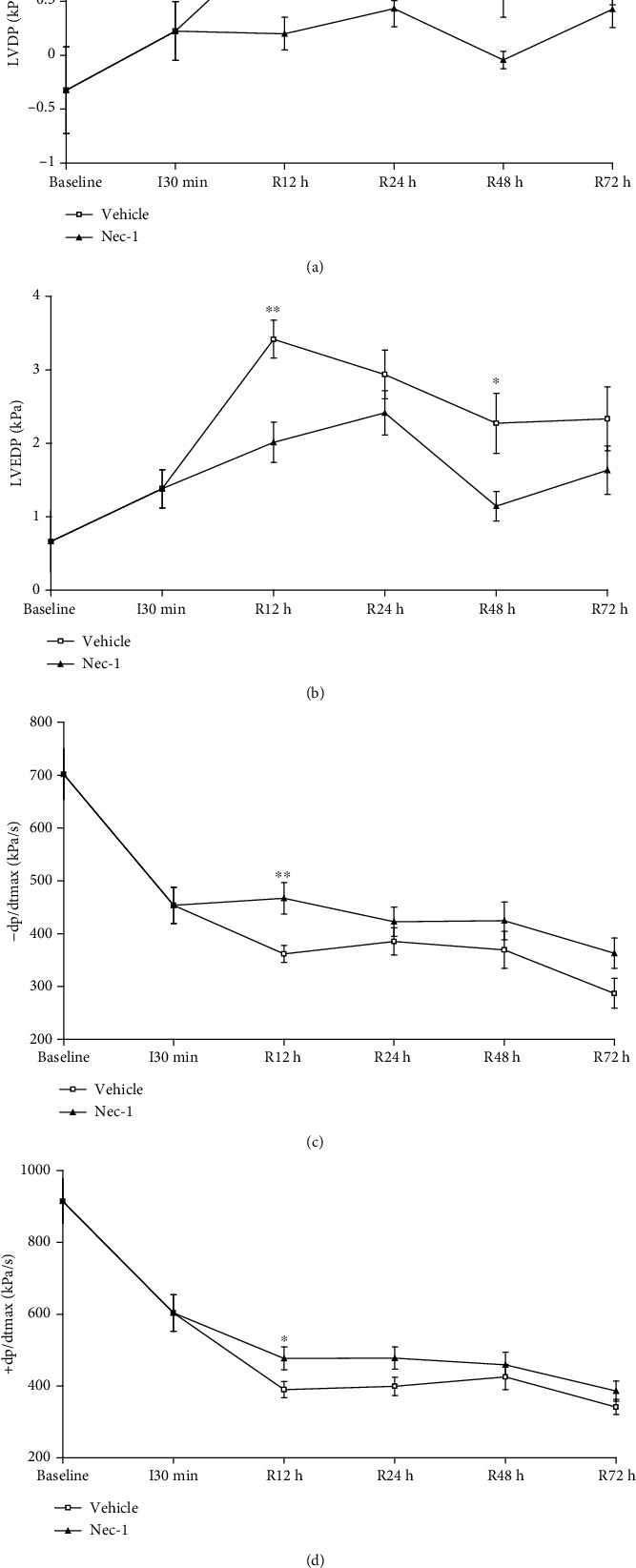
Assessment of cardiac function in vehicle and Nec-1-treated rats at different time points (12 h, 24 h, 48 h, and 72 h) during MI/R: (a) LVDP; (b) LVEDP; (c) −dp/dtmax; (d) +dp/dtmax in different groups. *n* = 8 mice for baseline; for vehicle group: I30min *n* = 10, R12h *n* = 10, R24h *n* = 10, R48h *n* = 10, R72h *n* = 10; for Nec-1 group: I30min *n* = 10, R12h *n* = 10, R24h *n* = 10, R48h *n* = 10, R72h *n* = 10. All data were expressed as mean ± SD. ^##^*P* < 0.01, baseline vs. vehicle group (I30 min). ^∗^*P* < 0.05 or ^∗∗^*P* < 0.01, Nec-1 group vs. vehicle group (I30min/R12h, 24 h, 48 h, 72 h).

Consistent with previous studies, myocardial ischemia and reperfusion resulted in a significant decrease in cardiac function (Figure 3). As shown in Figure 3, treatment with Nec-1 markedly improved LVDP, LVEDP, −dp/dtmax, and +dp/dtmax (Figures 3(a)–3(d)). Specifically, the LVDP and LVEDP (each at 12 and 48 h after reperfusion) of rats treated with Nec-1 at the onset of reperfusion were significantly lower than those of rats treated with the vehicle (Figures 3(a) and 3(b)); −dp/dtmax and +dp/dtmax (each at 12 h after reperfusion) of rats treated with Nec-1 at the onset of reperfusion were significantly higher than those of rats treated with the vehicle (Figures 3(c) and 3(d)). Notably, augmented LVDP and LVEDP in the vehicle group likely result from alterations in myocardial compliance due to the larger area of myocardial infarction. Treatment with Nec-1 can markedly diminish the alteration.

### 3.3. The Changes of LC3*β* at Different Time Points in the WB Assay

**Figure 4:**
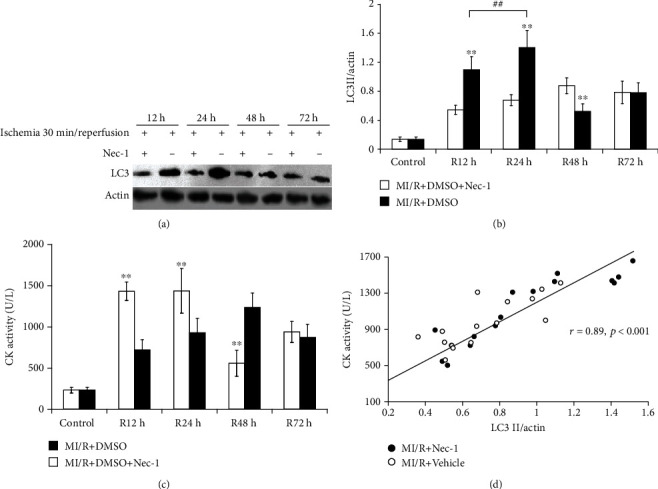
Detection and analysis of correlation between expression of MAP-LC3*β* in infarct zone and serum CK levels at different time points (12 h, 24 h, 48 h, and 72 h) during MI/R. (a) Representative immunoblot of LC3*β* protein in infarct zone (the tissue of the infarct zone is pale) of vehicle and Nec-1-treated rats at different time points (12 h, 24 h, 48 h, and 72 h) during MI/R. (b) Expression of LC3*β* protein in the infarct zone of vehicle and Nec-1-treated rats at different time points (12 h, 24 h, 48 h, and 72 h) during MI/R. Each column represents the ratio of the gray value of LC3*β* to IOD (actin). ^∗∗^*P* < 0.01, Nec-1 group vs. vehicle group. ^##^*P* < 0.01, Nec-1 group (R12h) vs. Nec-1 group (R24h). (c) Measurement of serum CK levels on rats subjected to 12 h, 24 h, 48 h, and 72 h reperfusion after myocardial ischemia for 30 min. Comparing the difference between the Nec-1 group and the vehicle group at different time points. Each column represents mean ± SD. ^∗∗^*P* < 0.01, Nec-1 group vs. vehicle group. (d) Relationship between LC3*β* immunoreactivity (% control) and serum CK levels (U/L) at different time points (12 h, 24 h, 48 h, and 72 h) during MI/R. Analysis of correlation shows that LC3*β* immunoreactivity was highly correlated with serum CK levels (*r* = 0.89, *P* < 0.001). All data were expressed as mean ± SD, *n* = 8 mice for baseline; for the vehicle group: I30min *n* = 10, R12h *n* = 10, R24h *n* = 10, R48h *n* = 10, R72h *n* = 10; for the Nec-1 group: I30min *n* = 10, R12h *n* = 10, R24h *n* = 10, R48h *n* = 10, R72h *n* = 10.

Autophagy is a cellular degradation process responsible for the turnover of unnecessary or dysfunctional organelles and cytoplasmic proteins. In the process, cytoplasmic proteins or dysfunctional organelles are sequestrated in a double-membrane-bound vesicle, termed autophagosome, delivered to the lysosome by fusion as an autolysosome and then degraded [25]. LC3*β* was localized in the autophagosome or autolysosome membrane and identified as an excellent marker of autophagic activity [13, 18–20, 26]. Through determining myocardial levels of LC3*β* at different time points after I/R *in vivo*, we found that the expression levels of LC3*β* at all time points after I/R were significantly upregulated compared to the control group. Treatment with Nec-1 resulted in a marked reduction in expression levels of LC3*β* at 12 and 24 h after reperfusion but had no significant changes at 48 and 72 h after reperfusion, compared with the relative vehicle group (Figures 4(a) and 4(b)). And interestingly, expression levels of LC3*β* in the Nec-1 group were significantly upregulated compared with the vehicle group at 48 h after reperfusion. This result may explain why the number of autophagosomes observed under the transmission electron microscope at 48 h after reperfusion in the Nec-1 group was higher than in the relative vehicle group.

### 3.4. The Results of CK Activity after Treatment with Nec-1

As an essential indicator of myocardial damage, serum CK levels were monitored at different time points after I/R. Treatment with Nec-1 significantly reduced serum CK levels at 12 and 24 h after reperfusion, compared with the relative vehicle group (722 ± 111 and 929 ± 271 U/L versus 1,433 ± 124 and 1,438 ± 174 U/L, *P* < 0.01 and *P* < 0.05, respectively), while CK levels in the control group were 232 ± 34 U/L (Figure 4(c)). However, treatment with Nec-1 could not diminish serum CK levels at 48 and 72 h after reperfusion; inversely, serum CK levels of the Nec-1 group at 48 h after reperfusion had markedly increased, compared with those of the relative vehicle group (559 ± 175 versus 1,238 ± 158, *P* < 0.01).

Because the trend of immunoblotting showed a striking similarity to the serum CK levels, in which Nec-1 reduced the value less than 12 h after reperfusion and delayed the peak to 48 h after reperfusion, it was decided to investigate the correlation between them. The results showed that, although these parameters of MI/R existed with interindividual variation, the expression of MAP-LC3*β* was significantly correlated with the serum CK levels (*r* = 0.89, *P* < 0.001, Figure 4(d)). These results demonstrated that the necroptosis induced by MI/R delayed autophagy-like cell death and played a vital role in MI/R.

## 4. Discussion

This study demonstrated that Nec-1 (0.6 mg/kg) could maintain myocardial ultrastructural integrity and markedly reduce myocardial infarct size and reactive fibrosis at late reperfusion after MI. Additionally, LVDP and LVEDP, as essential determinants of LV remodeling, were also significantly lower in the Nec-1 group than in the vehicle group. Thus, we confirmed that Nec-1 (0.6 mg/kg) could protect the myocardium against I/R injury in rats by reducing the loss of myocardial cells and LV remodeling. Moreover, it also found that Nec-1 at higher concentrations (1.8 mg/kg) would increase the mortality rate of rats subjected to chronic myocardial ischemia (data not shown). The results indicated that Nec-1 was cardioprotective at lower concentrations but had harmful actions with increased concentrations, which appears to coincide with the report of C. C. Smith et al. [24].

Another interesting work was to determine the feature of necroptosis in MI/R. Autophagy is a highly conserved cellular mechanism of protein recycling that may lead to programmed cell death (type II programmed cell death) [11, 12, 14, 27]. Therefore, autophagy plays a dual role as a cell-survival pathway and as an intrinsic cell death mechanism under some circumstances. As a terminally differentiated cell, the cardiomyocyte is thought to be more sensitive to autophagy than other cell types. Some studies have shown that autophagy is a protective mechanism in chronic ischemia [20, 21], and inhibiting autophagy resulted in a significant aggravation of cardiomyocytes in I/R injury. Inversely, enhancing autophagy has an underlying protective response against I/R injury in heart cells [20]. Recently, the effect of autophagy in mediating cell survival and death in MI/R injury is still controversial [26]. It has been considered that the upstream mechanism of autophagy could be a critical event in mediating cell survival and death during ischemia and reperfusion in the heart. However, autophagy is considered as the end of many paths [27, 28]; thus, it is difficult to judge which pathway will be essential.

In 2005, L. Yan et al. discovered a small molecule called Necrostatin-1 (Nec-1), which can inhibit necroptosis, a nonapoptosis pathway characterized by autophagy, and considered autophagy as a downstream consequence of necroptosis rather than a contributing factor to necroptotic cell death in cerebral I/R injury [18]. Because both necroptosis and autophagy are mechanisms of delayed ischemic brain injury, we observed the alterations of LC3*β* expression levels and serum CK levels, respectively, at 12, 24, 48, and 72 h reperfusion after MI, and found that the administration of Nec-1 at the onset of reperfusion significantly reduced the release of creatine kinase and downregulation of autophagy at 12 and 24 h after reperfusion, but the effects were not sustained to 48 and 72 h after reperfusion. A correlation exists between the release of creatine kinase and the expression of autophagy. Administering Nec-1 to coincide with reperfusion could decrease and delay the peak to 48 h after reperfusion. These data are similar to those of Zhao et al., who demonstrated that infarct size increased to a peak at 24 h of reperfusion with no further increase at 48 and 72 h of reperfusion [29], consistent with the peak of autophagic activity. This evidence provides strong support for our hypothesis that necroptosis is not only an autophagy-like cell death but is also an essential contribution to delayed reperfusion injury after myocardial ischemia.

This study confirmed that the myocardial protective effect of Necrostatin-1 in rat myocardial I/R injury is related to autophagy. However, the effective dose of the drug and the related pharmacological mechanism are not clear. To further determine the effect of Nec-1, additional research can explore the lowest effective dose, the best therapeutic dose and side effects of the drug, and the molecular biological mechanisms related to Nec-1.

In summary, this research demonstrated that the administration of Nec-1 inhibited an autophagy-like cell death that may play an important role during delayed reperfusion injury after myocardial ischemia, and it significantly improved cardiac function after myocardial ischemia and reperfusion, suggesting that antinecroptosis treatment may improve the clinical outcomes of patients with ischemic heart disease. However, the mechanism of autophagy-like cell death needs to be investigated further. Thus, in the following study, Nec-1 of binding targets will be identified, and other therapeutic strategies explored to reduce myocardial cell death responsible for myocardial I/R injury.

## Data Availability

The datasets used and analyzed during the current study are available from the corresponding author on reasonable request.
